# PyMUS: Python-Based Simulation Software for Virtual Experiments on Motor Unit System

**DOI:** 10.3389/fninf.2018.00015

**Published:** 2018-04-11

**Authors:** Hojeong Kim, Minjung Kim

**Affiliations:** Convergence Research Institute, Daegu Gyeongbuk Institute of Science and Technology, Daegu, South Korea

**Keywords:** motoneuron, muscle fibers, motor unit, python, computer modeling, simulation software

## Abstract

We constructed a physiologically plausible computationally efficient model of a motor unit and developed simulation software that allows for integrative investigations of the input–output processing in the motor unit system. The model motor unit was first built by coupling the motoneuron model and muscle unit model to a simplified axon model. To build the motoneuron model, we used a recently reported two-compartment modeling approach that accurately captures the key cell-type-related electrical properties under both passive conditions (somatic input resistance, membrane time constant, and signal attenuation properties between the soma and the dendrites) and active conditions (rheobase current and afterhyperpolarization duration at the soma and plateau behavior at the dendrites). To construct the muscle unit, we used a recently developed muscle modeling approach that reflects the experimentally identified dependencies of muscle activation dynamics on isometric, isokinetic and dynamic variation in muscle length over a full range of stimulation frequencies. Then, we designed the simulation software based on the object-oriented programing paradigm and developed the software using open-source Python language to be fully operational using graphical user interfaces. Using the developed software, separate simulations could be performed for a single motoneuron, muscle unit and motor unit under a wide range of experimental input protocols, and a hierarchical analysis could be performed from a single channel to the entire system behavior. Our model motor unit and simulation software may represent efficient tools not only for researchers studying the neural control of force production from a cellular perspective but also for instructors and students in motor physiology classroom settings.

## Introduction

A motor unit comprises a single motoneuron and the muscle fibers innervated by that motoneuron and is the smallest functional element of all movements (Heckman and Enoka, 2012). Spinal motoneurons receive both excitatory and inhibitory synaptic inputs from upper motor centers in the brain and peripheral sensory organs. Muscle fibers transform the neural signals from motoneurons into force output during movements. To accurately understand the neural mechanisms controlling muscle contraction and force production, a systematic investigation of the input-output properties of motor units at the system level, including both motoneurons and muscle subunits, is crucial due to the non-linearities inherent in motoneuron and muscle behavior. Thus, a physiologically plausible computational model and simulation software are urgently needed to efficiently investigate complex interactions between motoneurons and muscle fibers in neuromuscular systems.

Motor unit models have been developed under static and dynamic conditions. The static motoneuron models have been used to represent the input–output relationship of motoneurons at steady state (Heckman and Binder, 1991, 1993; Fuglevand et al., 1993). Since those models have been phenomenologically formulated it has been difficult to expand them to reflect the non-linear properties experimentally observed from the motor unit system. In contrast, the dynamic motoneuron models have been mechanistically formulated to investigate the influence of biophysical properties of motoneurons on non-linear input-output behavior of motor unit system using reduced modeling approach (Elias et al., 2012; Powers and Heckman, 2017). However, there has been a lack of a suitable method for determination of model parameters to reflect key cell type associated properties (i.e., input resistance, time constant, rheobase current, afterhyperpolarization duration and dendritic signal attenuation) in the previous dynamic models (Kim, 2017a). Furthermore, in all previous studies the muscle unit has been represented using a simplified second-order mechanical system exclusively for isometric contractions. In addition, the simulation software providing graphical user interfaces have been developed separately for nervous (Schutter, 1992; Brette et al., 2007; Muller et al., 2015) and muscular (Cheng et al., 2000; Delp et al., 2007) systems. To this end, the existing model and software for the motor unit need to be improved to incorporate cell type specific properties and non-linear behaviors allowing for integrative investigations on the motor unit system under a broad range of physiological input conditions.

Applying a computational analysis, the underlying mechanism of the non-linear behavior of spinal motoneurons has been successfully identified by testing an experimental hypothesis. Since the discovery of persistence inward current (PIC) during voltage clamp experiments (Schwindt and Crill, 1980), the non-linear input-output relationship of motoneurons has been intensively investigated in many species, including turtles (Hounsgaard and Kiehn, 1989), mice (Carlin et al., 2000), rats (Bennett et al., 2001) and cats (Lee and Heckman, 1998a). In the presence of monoamines, during normal behaviors, motoneurons display bistable behavior in which the firing state transitions from quiescence to regular firing or from low-frequency to high-frequency stable firing following the activation of PICs by a brief excitatory pulse input. This bistability has also been shown to lead to the production of counterclockwise hysteresis in the motoneuron input-output relationship during the slowly ascending and descending current stimulation at the soma (Hounsgaard et al., 1986; Lee and Heckman, 1998b). The underlying mechanism of this non-linear input–output process has been investigated in both experimental and computational studies, and the persistent inward current-generating channels in the dendrites have been shown to be critical for the production of the bistable and hysteretic firing behaviors of motoneurons (Hounsgaard and Kiehn, 1993; Ballou et al., 2006). The PIC channels must be clustered over the dendritic regions and separated from the soma by 0.3 – 0.8 mm (Carlin et al., 2000; Elbasiouny et al., 2005; Bui et al., 2006). Furthermore, the type-specific electrical properties of motoneurons, such as input resistance, membrane time constant and signal attenuation properties over the passive dendrites, have also been shown to be fundamental determinants that modulate the effects of dendritic PIC activation on the input-output processing of motoneurons (Kim, 2017a). In summary, the dendritic PIC location and type-specific properties of motoneurons are primary factors that determine the non-linear firing behavior of motoneurons.

The dependencies of muscle activation on neural signals from motoneurons and the length variation during movements have also been extensively investigated using both experimental and computational approaches. Under full-excitation conditions, the force-length (F-L) and force-velocity (F-V) relationships are the fundamental properties of muscle units (Gasser and Hill, 1924; Hill, 1970; Zajac, 1989; Brown et al., 1996). The bell-shaped F-L relationship represents the optimal length at which the peak force is produced as the muscle length increases under isometric (constant-length) conditions. The F-V relationship represents the velocity dependency of force degradation during the shortening of muscle length and force potentiation during the lengthening of muscle length under isokinetic (constant-velocity) conditions. Based on these fundamental input-output properties, the muscle has been modeled as a mechanical system known as the Hill-type model, and this model includes a contractile element representing the muscle fibers, serial elastic elements representing the tendon organs and parallel elastic and viscous elements representing the connective tissues surrounding the muscle fibers (Zajac, 1989). In the Hill-type muscle, the peak force at the optimal length during an isometric contraction under full excitation is scaled proportionally by three factors (muscle activation level and F-L and F-V relationships) ranging from 0 to 1, while the stimulation rate and muscle length vary over time. However, this modeling approach does not sufficiently reflect the overall input–output properties of a muscle unit for the full physiological range of stimulation rates and muscle lengths (Sandercock and Heckman, 1997; Millard et al., 2013). In particular, under the physiological range (<20 Hz) of stimulation rates, the muscle force for shorter-than-optimal muscles is much smaller than that for longer-than-optimal muscles during isometric contractions. During locomotor-like movements, the force under a low-frequency (<20 Hz) stimulation rate is much more degraded than that under a high-frequency stimulation rate (Perreault et al., 2003). In summary, the complex interactions among the sarcoplasmic calcium dynamics, cross-bridge formation and length variation should be considered to realistically model the force production of muscle units over the full physiological range of stimulation rates and muscle lengths.

First, we constructed a computationally efficient physiologically plausible model of motor units. To model the motoneuron, we used a newly developed reduction modeling approach in which the reduced motoneuron could reflect the key system properties that determine the non-linear input–output processing of real motoneurons (Kim et al., 2014). We modeled the force production of muscle units using a recently developed muscle-tendon modeling approach that matches a wide range of experimental measurements obtained from cat soleus muscles, including the force production during twitch, sub-tetanic and tetanic contractions under both static and dynamic variations in muscle length, and the length- and velocity-tension properties under full muscle activation (Kim et al., 2015). Then, we developed a simulation software for systematic investigations and analyses in the Python development environment and validated the simulation capability and functionality of this software by reproducing the simulation results obtained using existing simulation software under physiological conditions. It is notable that the current version of the simulation software was designed to operate based solely on graphical user interfaces for easy use so that the flexibility in extending the model and simulation by the user may be limited in comparison to script-based simulation software.

## Materials and Methods

### Canonical Modeling of a Motor Unit

**FIGURE 1:**
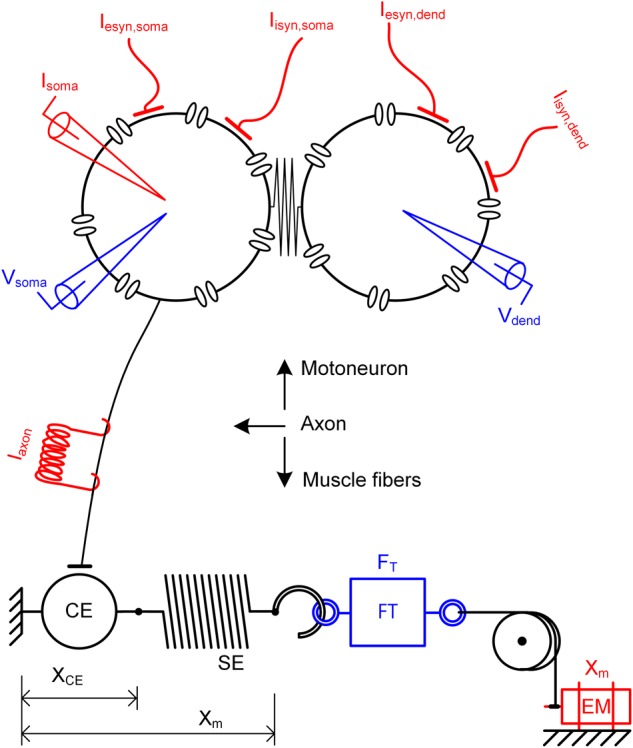
Schematic diagram of a model motor unit system. Reduced motoneuron model consisting of somatic and dendritic compartments with coupling conductance **(upper)**, axon model for the perfect transmission of neural signals from the motoneuron **(middle)** and muscle-tendon model consisting of a contractile element (CE) and serial elastic element (SE) in series **(bottom)**. Input signals (red) to the motor unit model include the intracellular current injection at the soma (I_soma_), excitatory and inhibitory synaptic inputs at the soma (I_esyn,soma_ and I_isyn,soma_) and the dendrite (I_esyn,dend_ and I_isyn,dend_), current impulse stimulation over the axon (I_axon_) and length variation at the muscle (X_m_). Output signals (blue) from the motor unit model include the membrane voltage at the soma (V_soma_) and the dendrites (V_dend_) and force at the muscle (F_T_). X_CE_, FT and EM indicate the length of the contractile element, force transducer and electrical motor, respectively.

A motor unit comprises a single motoneuron and the set of muscle fibers (or muscle unit) innervated by the motoneuron through its axon. In this study, the model motor unit was constructed by coupling two subunit models of a motoneuron and muscle unit to the axon model (see **Figure 1** for a schematic diagram). The motoneuron and muscle unit were biophysically modeled by applying the recently developed reduction method, which allowed all model parameters to be directly constrained from the key system properties that are experimentally measurable in real motoneurons (Kim et al., 2014) and muscle-tendon complexes (Kim et al., 2015). The reduced motoneuron model consisted of two compartments (i.e., one component included the soma, axonal hillock and initial segment and the other compartment included the dendrites) coupled with an electrical conductance (upper panel in **Figure 1**). The point model of a muscle unit is composed of a contractile element representing the muscle fibers and a serial elastic element representing the tendon (bottom panel in **Figure 1**). The axon was modeled to simply represent the perfect transduction of neural signals over the axonal nerve with a single parameter of conduction delay time from the motoneuron to the muscle unit (middle panel in **Figure 1**).

#### Electrical Behavior of Motoneurons

##### Passive dynamics

In our conductance-based two-compartment modeling framework, the passive dynamics of a motoneuron are captured using the following five cable parameters: two specific membrane conductances (G_m,S_ and G_m,D_) at the somatic and dendritic compartments, two specific membrane capacitances (C_m,S_ and C_m,D_) at the somatic and dendritic compartments, and one coupling conductance (G_C_) between the somatic and dendritic compartments. In a top–down manner, all five cable parameters are analytically constrained simultaneously to retain the following five system properties: two whole-cell properties [input resistance (R_N_) and membrane time constant (τ_m_)] measured at the soma and three signal propagation properties over the dendrites for direct current (DC) signals flowing between the soma and the dendrites and alternating current (AC) signals transferred from the soma to the dendrites (see Kim et al., 2009; Kim and Jones, 2012 for details). These DC and AC signal transductions over the dendrites are represented by the voltage attenuation (VA) factor, which is defined as the ratio of the voltage at the measurement site to the voltage at the stimulation site. In this study, the three VA factors, i.e., the soma-to-dendrite VA with DC input at the soma, the soma-to-dendrite VA with AC input at the soma and the dendrite-to-soma VA with DC input to all points of the dendrite, are referred to as VA_SD_^DC^, VA_SD_^AC^ and VA_DS_^DC^, respectively.

##### Active mechanisms

All active membrane properties considered in previous computational studies investigating spinal motoneurons were incorporated in the present version of the simulation software (Booth et al., 1997; Hornby et al., 2002; Powers et al., 2012; Elbasiouny, 2014). The rhythmic firing activity of motoneurons was produced at the somatic compartment via interactions between the following six active currents: fast sodium (I_Na,f_), delayed rectifier potassium (I_K,dr_), N-type calcium (I_Ca,N_), calcium-dependent potassium (I_K(Ca)_), persistent sodium (I_Na,p_) and hyperpolarization-activated mixed-cation (I_H_) currents. The rheobase current and afterhyperpolarization duration of an action potential could be separately modulated by varying the conductance [i.e., G_Na,f_ in Equation (1.8) in the Appendix] for I_Na,f_ and the percentage [i.e., f_S_ in Equation (1.11) in the Appendix] of free to bound Ca^2+^ ions in the soma, respectively (Kim, 2017a). The plateau potentials and voltage oscillations at the dendritic compartment were produced using the following seven active currents: fast sodium (I_Na,f_), delayed rectifier potassium (I_K,dr_), N-type calcium (I_Ca,N_), calcium-dependent potassium [I_K(Ca)_], persistent sodium (I_Na,p_), hyperpolarization-activated mixed-cation (I_H_) and low-voltage-activated L-type calcium (I_Ca,L_) currents. The dynamics of the calcium concentration were also considered to update the I_K(Ca)_ (Booth et al., 1997) and equilibrium potential of the calcium current (E_Ca_) (McIntyre and Grill, 2002) in both the somatic and dendritic compartments. The Hodgkin-Huxley type formulation was applied for modeling the voltage-gated ion currents: I_Na,f_, I_K,dr_, I_Ca,N_, I_Na,p_, I_Ca,L_ (McIntyre and Grill, 2002) and I_H_ (Powers et al., 2012). The system equations used to generate the reduced motoneuron model, including all transmembrane ionic currents and calcium dynamics, are presented in full in the Appendix.

#### Force Production of Muscle Fibers

The multiple signal transformations that occurred in the sarcomere for force production are represented by the three sub-modules reported in a previous study (Kim et al., 2015). In this modular muscle modeling approach, model parameters could be constrained separately based on experimental data obtained for individual sub-modules.

##### Module 1

The first module represents the transformation of the spike signals from the motoneurons into the concentration dynamics of calcium (Ca) and Ca bound to troponin (CaT) in the sarcoplasm (SP). The dynamics of the Ca and CaT concentration in the SP were modeled by simplifying the chemical reactions experimentally identified in single mouse muscle fibers (Westerblad and Allen, 1994). The sarcoplasmic Ca concentration was determined by the release (R) and reuptake (U) of Ca through the membrane of a sarcoplasmic reticulum (SR) containing free Ca and calsequestrin (CS), and the interactions with free Ca-buffering proteins (B) and troponins (T) in the SP.

##### Module 2

The second module addresses the conversion of the CaT concentration in the SP to the level of muscle activation [A(t)]. This conversion was modeled based on the non-linear (i.e., sigmoidal) relationship between the Ca concentration in the SP and force production that has been experimentally observed under steady-state conditions, reflecting the cooperativity in cross-bridge formation (Shames et al., 1996). In this module, the steady-state relationship of Ca-force was first mapped to that of CaT-activation level (Ã_∞_) for the steady Ca stimulation. The dynamics of Ã was further represented applying the Morris–Lecar formulation that has been used to mathematically represent the dynamics of gating variables underlying membrane excitability (Morris and Lecar, 1981). Then, the Ã(t) was updated to the A(t) in the exponential form [Ã(t)]^α^, where the exponent α represents the transient variation in the sarcoplasmic Ca concentration during neural excitation.

##### Module 3

Considering the length- and velocity-tension properties, the third module simulates the transformation of A(t) into the muscle force based on Hill-type muscle mechanics consisting of contractile element (CE) and serial elastic element (SE) lumping tendon and aponeurosis compliance. The force output was determined by multiplying the stiffness of SE by the difference between the deviation in the total muscle (X_m_) and contractile element length (X_CE_) from their initial lengths. The X_CE_ was determined at various muscle lengths and levels of activation using the modified Hill-Massima equations to represent the velocity-tension relationship for both muscle shortening and lengthening at various levels of muscle length and activation. All four coefficients [i.e., a_0_-d_0_ in Equation (2.7) in the Appendix] in the Hill-Massima equations could be analytically determined using the inverse equations against the velocity-tension curve experimentally characterized (Sandercock and Heckman, 1997) (see the Methods in Kim et al., 2015 for details). All experimentally identified dependencies of soleus muscle activation dynamics on muscle length during isometric contractions were incorporated into the present muscle unit model by making the rate constant [i.e., K5 in Equation (2.5) in the Appendix] a function of the muscle length in the first module and embedding the length-tension relationship of the soleus muscle in the third module. The velocity-tension relationship of the muscle unit (or muscle fibers innervated by a single motoneuron) was scaled by multiplying two coefficients [i.e., a_0_ and c_0_ in Equation (2.7) in the Appendix] in the Hill-Massima equations by the ratio of the peak force of the muscle unit to the peak force of the entire muscle at the optimal muscle length during an isometric contraction under full excitation.

The equations used to generate the individual sub-modules are fully presented in the Appendix.

### Considerations for the Motor Unit Simulation

#### Topology and Geometry

##### Motoneuron

The morphological characteristics of the motoneuron dendrites and the cable properties determine the electrotonic structure of the dendrites depending on both the direction and type of electrical signals propagating between the soma and the dendrites (Carnevale and Johnston, 1982; Zador et al., 1995). In our reduced modeling approach, the complex motoneuron structure is collapsed into two compartments (i.e., somatic and dendritic). The electrotonic structure of the motoneuron dendrites is physiologically reflected between the somatic and dendritic compartments, allowing all cable parameters (i.e., G_m,S_, G_m,D_, G_C_, C_m,S_, and C_m,D_) of the reduced motoneuron to be analytically determined simultaneously to retain the three voltage attenuation factors (i.e., VA_SD_^DC^, VA_DS_^DC^, and VA_SD_^AC^) calculated along the paths of the dendritic trees as a function of the path length from the soma (Kim et al., 2014). The voltage attenuation factors between the soma and a single point over the dendrites (i.e., point-to-point condition) (Zador et al., 1995; Bui et al., 2003) or between the soma and all points over dendrites at the same path length (i.e., point-to-all condition) have been characterized (Kim et al., 2014; Kim, 2017a). In the current version of the software, the direction- and type-dependent signal propagation properties over passive motoneuron dendrites can be simulated based on the physical length by directly varying the individual voltage attenuation factors between 0 and 1. The passive dendritic excitability (i.e., dendritic input resistance) is also physiologically incorporated into the reduced modeling framework by the formulation of the somatic input resistance (i.e., R_N_) multiplied by the asymmetry ratio (i.e., VA_SD_^DC^/VA_DS_^DC^) of the dendritic signal propagation as reported in a previous study (Kim and Jones, 2011). Furthermore, the surface area of the dendrites could be modulated by adjusting a model parameter (p) that indicates the ratio of the surface area of the somatic compartment to the surface area of the dendritic compartment (Kurian et al., 2011; Kim and Heckman, 2015).

##### Muscle unit

The muscle unit was modeled by assuming that the force production of individual muscle fibers is scalable from that of the sarcomere and that the muscle fibers comprising a muscle unit are uniformly arranged with identical lengths within the muscle. Under these assumptions, the present muscle unit model represents the average force production of all muscle fibers innervated by a single motoneuron. In this study, the force production of a single muscle unit was, via simulation, scaled from the maximum force that the entire muscle may produce during an isometric contraction at the optimal muscle length under full excitation.

#### Channel Selection and Distribution

##### Motoneuron

The types of active transmembrane channels may differ depending on the location over the soma and dendrites. This heterogeneous distribution of voltage-gated ion channels was simulated by selecting a specific set of voltage-gated ion channels for each compartment in the reduced modeling framework. Furthermore, the variation in the physical location of the dendritic voltage-gated ion channels was simulated by changing the physiological values of the three voltage attenuation factors that were calculated as a function of the path length along the dendrites from the soma in anatomically reconstructed motoneurons under the point-to-point condition (Kim et al., 2009; Kim and Jones, 2012) and the point-to-all points condition (Kim et al., 2014).

##### Muscle unit

In this study, the transduction of neural signals from the motoneuron to the muscle unit at the neuromuscular junction was assumed to be perfect. The action potentials from the reduced motoneuron are directly transferred to the muscle unit with a time delay reflecting the conduction velocity of the axonal nerve. In response to the neural signals from the reduced motoneuron, the release and uptake of calcium ions through the membrane of the sarcomere reticulum at the muscle unit are phenomenologically simulated and modulated by two time constants (i.e., τ_1_ and τ_2_) for Ca release and one rate constant (i.e., K) for Ca reuptake [see Equation (2.5) in the Appendix].

#### Stimulation Protocols for Motoneurons

Both intracellular stimulations and synaptic inputs are considered in this study. Regarding the intracellular stimulation, the following two types of stimulation protocols are experimentally applied at the soma (Hounsgaard et al., 1988; Lee and Heckman, 1998b): (1) a long-lasting current step along with alternating excitatory and inhibitory current pulses to test the cell excitability at the steady-state and (2) a current ramp that monotonically rises and falls to simulate the input–output relationship at a wide range of stimulation intensities. Regarding the synaptic inputs, both excitatory and inhibitory synaptic inputs were applied at the soma and dendrites. Similar to the intracellular stimulation, the synaptic conductances were modulated in the form of a long-lasting step along with alternating pulses and an ascending and descending triangular shape over time. Synaptic currents with noise were also selectively incorporated into the synaptic conductance to reflect the noisy background activity and synaptic transmission (Destexhe and Pare, 1999). In the simulation of synaptic noise, the noise amplitude increased in proportion to the maximum conductance of the synaptic input (Powers et al., 2012). The method used to generate the synaptic currents with noise has been fully described in the previous study (see the Appendix for equations). For the axonal stimulation, a supra threshold current impulse sufficient to evoke a spike over the axon was generated at both regular and irregular frequencies. The current impulses at an irregular (or random) frequency were produced from a normal (Gaussian) distribution given a mean frequency and standard deviation. Furthermore, input signals that are not provided by the current version of the software may be defined by the user and imported directly from data files.

#### Variation in Muscle Length

The muscle length was changed over the full physiological range from the minimum of -16 mm to the maximum of 0 mm with the optimal muscle length of -8 mm based on experimental studies investigating cat soleus muscles (Sandercock and Heckman, 1997). In this study, the following three types of length variations were considered: (1) a constant length under steady-state conditions (isometric), (2) a constant change in muscle length over time (isokinetic) and (3) locomotor-like movement (dynamic). Under the dynamic condition, the locomotor-like movement was generated via random variation of muscle length produced at a bandwidth ranging from 0 to 5 Hz, which matched the changes observed in the soleus length during unrestrained locomotion (Goslow et al., 1973). The dynamic variation in the muscle length over time was calculated using the following two steps: random numbers were created in a white uniform 10-kHz sequence using a random number generator and then filtered with a low-pass FIR filter using a Blackman window. All length perturbations were centered around an operating point -8 mm less than the physiological maximum length (0 mm). Furthermore, length signals that are not provided by the current version of the software may be defined by the user and imported directly from data files.

#### Numerical Integration

The accuracy, stability, and performance of the computer simulation were considered in selecting the optimal method for the numerical integration of the model motoneuron, muscle fibers and motor unit. Among the ordinary differential equation (ODE) solvers available in the Python library (i.e., scipy), VODE and LSODA were the best options for our motoneuron and muscle unit models, respectively. Although the simulation with LSODA was faster than that with VODE for both the motoneuron and muscle unit models, the LSODA solver caused an unstable simulation and inaccurate integration in the motoneuron model during the excitatory and inhibitory current pulse injections over a short period of time (i.e., 300 ms) at the somatic compartment. The simulations using other methods (i.e., dopri5 and dop853) were much slower for all three models (i.e., motoneuron, muscle unit and motor unit). For the stochastic simulations, including the noise in the synaptic conductance, VODE and LSODA were also applied to numerically solve the motoneuron and muscle unit models.

### Considerations for Software Design

#### Structure

**FIGURE 2:**
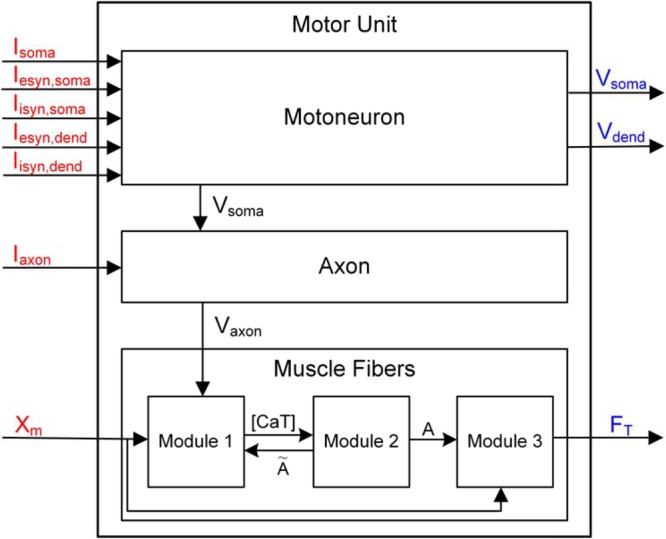
Data flow diagram for PyMUS. Input and output signals considered for the simulation software are indicated in red and blue as represented in **Figure 1**. V_soma_ and V_axon_ represent the membrane voltage data flowing from the motoneuron to the axon and from the axon to the muscle fibers, respectively. [CaT], Ã and A in the muscle fibers indicate the concentration of Ca^2+^ bound to troponin in the sarcoplasm, muscle activation level for steady and dynamic excitation condition, respectively (see Force Production of Muscle Fibers in “Materials and Methods” for the detailed explanation on the three sub-modules).

The design of the overall software structure enabled electrophysiological experiments at both the single-cell and motor unit levels (**Figure 2**). The three components generally involved in electrophysiological experiments investigating the motor unit system were identified as the input signal, target system and output signal. The input signal for the motoneuron includes the intracellular and synaptic stimulations at the soma and synaptic stimulations at the dendrite, and for the muscle unit, the input signal includes the electrical stimulations at the axon and the variation in the muscle length. The target system included the motoneuron, muscle unit and motor unit. In this study, the output signal included the spikes at the soma and the dendrites in the motoneuron and the force from the muscle unit.

#### Functionality

**FIGURE 3:**
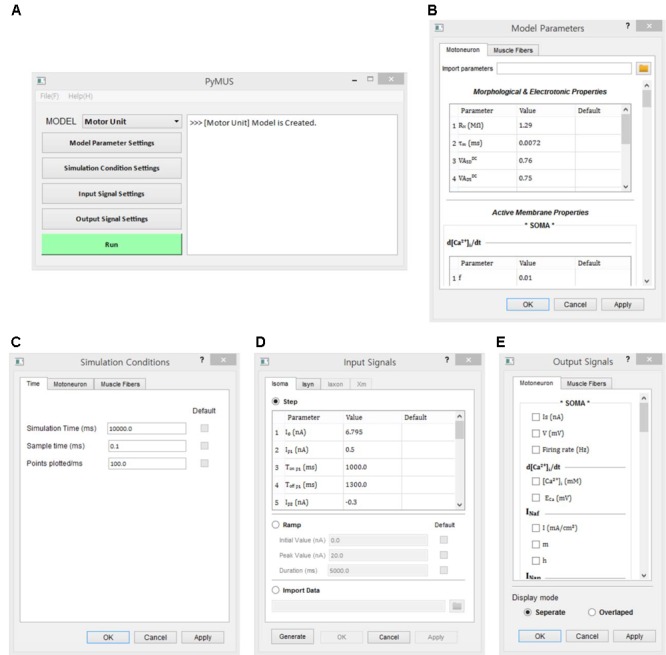
Graphical user interfaces for PyMUS. The main window **(A)** provides all graphical user interfaces for the model selection **(A)**, model parameter setup **(B)**, simulation condition setup **(C)**, input signal setup **(D)**, output signal setup for display of simulation results **(E)**.

The design of the simulation software allowed the simulations to be fully operated using a graphical user interface (GUI). The design of the GUI allowed for a generic computational procedure to be performed using modeling and simulation approaches (**Figure 3**). The main window consists of one state window and six buttons for model selection, parameter setting, simulation condition setting, input setting, output display setting and run control. Using the MODEL button, the target model to be simulated is selected among the motoneuron, muscle unit and motor unit models. The Model Parameters window, which pops up via the Model Parameter Settings button, provides the GUI interfaces for setting the model parameter values manually or automatically by importing pre-determined data into the software. The Simulation Conditions window is generated by clicking on the Simulation Condition Settings button and includes the GUI interfaces for setting the simulation time, display quality and initial values of the model equations. The Input Signals window is produced by clicking on the Input Signal Settings button and allows the type and protocol for injecting the input signals into the model to be selected. The Output Signals window, which pops up by clicking the Output Signal Settings button, enables the output variables to be displayed and plotting options to be selected. To efficiently compare the multiple output variables simultaneously, the design of the simulation software allows for the output variables to be selected in the Output Signals window and displays these variables either individually on separate panels or together on the same panel. To support offline analyses, the simulation data are also saved in a separate file. Through the Run button, the simulation can be started or stopped.

#### Extensibility

To enhance the software extensibility for future changes and improve the management and reuse of the software, the current version of the simulation software for the motor unit system was designed and implemented under the object-oriented paradigm (Bal and Grune, 1994) and reported using the UML 2.1 standard^1^.

#### Interoperability

To promote the interoperability of the simulation software between operating systems, the software was implemented in the open-source Python software environment (i.e., Python 2.7) using Anaconda (version 2.2.0) that is freely downloadable at https://repo.continuum.io/archive/ (Oliphant, 2007; Millman and Aivazis, 2011). Scipy libraries (version 0.15.1) were used to solve the models (Jones et al., 2001). The Pandas (version 0.15.2) and Numpy (version 1.9.2) libraries were used for data management (Mckinney, 2010; Van Der Walt et al., 2011). PyQt4 (version 4.10.4) and Matplotlib (version 1.4.3) were used for the GUI design and data plotting in the Python software environment (Hunter, 2007; Summerfield, 2008). The source code and binary files for the simulation software produced in this study are publically available for download from online repositories^2^.

## Results

### Implementation of the Simulation Software

After identifying the functional and non-functional requirements for the motor unit simulation software, the software was implemented using the standard UML language. The structural and dynamical aspects of the simulation software developed in this study were addressed by drawing a class diagram and state diagram, respectively.

#### Class Diagram

**FIGURE 4:**
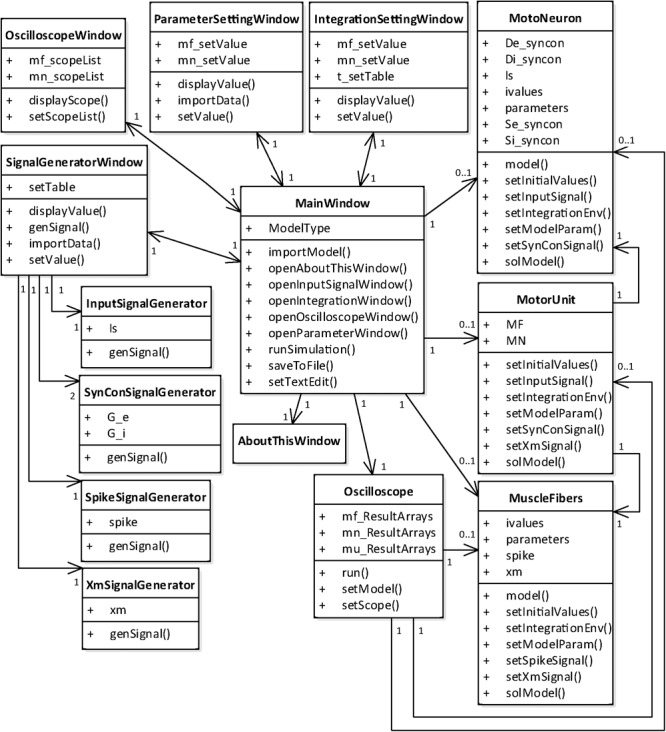
Class diagram for PyMUS. The MainWindow class is mutually associated with all classes for the graphical user interfaces (AboutThisWindow, ParameterSettingWindow, IntegrationSettingWindow, SignalGeneratorWindow and OscilloscopeWindow) and includes the classes for the model construction and simulation (Oscilloscope, MotoNeuron, MuscleFibers, and MotorUnit). The SignalGeneratorWindow class further includes four classes for generation of input signals (InputSignalGenerator, SynConSignalGenerator, SpikeSignalGenerator, and XmSignalGenerator). The MotoNeuron class, MuscleFibers class and MotorUnit class are selectively included in the MainWindow class depending on the model type selected by the users in the main window.

From a structural perspective, the simulation software consists of two groups of classes (**Figure 4**). One group was developed for the graphical user interfaces through which the users can set up and run the simulation and manage the simulation data. This class group includes six main classes for the generation of the main window and model selection (MainWindow class), setup of the model parameters (ParameterSettingWindow class), setup of the simulation conditions (IntegrationSettingWindow class), setup of the input signals (SignalGeneratorWindow class), setup of the output signals (OscilloscopeWindow class) and display of software information (AboutThisWindow class). The SignalGeneratorWindow class further includes four additional classes for the generation of intracellularly injected current inputs at the soma (InputSignalGenerator class), synaptic inputs at both the soma and dendrite (SynConSignalGenerator class), current impulse stimulation at the axon (SpikeSignalGenerator class) and muscle length variation in the muscle unit (XmSignalGenerator class). The other class group was developed for run control and data storage (Oscilloscope class) and model construction and numerical integration (MotoNeuron class for motoneuron, MuscleFibers class for muscle unit and MotorUnit class for motor unit). In our software, the MainWindow class has a relationship with all other classes, allowing users to access all classes through the main window.

#### State Diagram

**FIGURE 5:**
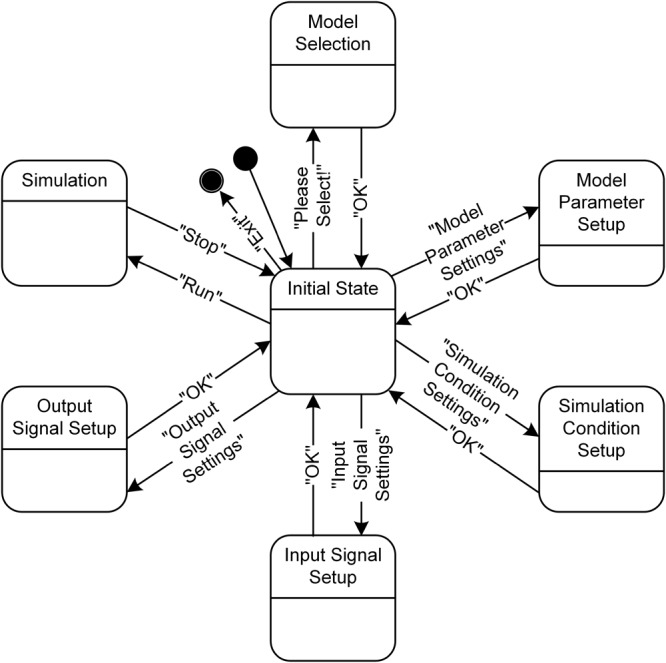
State diagram for PyMUS. PyMUS can transition back and forth between the initial state and the other main states (Model Selection, Model Parameter Setup, Simulation Condition Setup, Input Signal Setup, Output Signal Setup and Simulation) according to the event triggered by the button in the GUI window. The black filled circle without and with an additional black circle represent the start and end of the software execution.

From a dynamical perspective, the simulation software has seven main states (**Figure 5**). The individual states for the model selection, model parameter setup, simulation condition setup, input signal setup, output signal setup, and simulation can transition from and into the initial state according to the event triggered by users pushing the button in the GUI windows (see **Figure 3**). In addition, the four states, except for the Model Selection, Simulation and Initial State, can mutually transition among each other without going into the Initial State when the “Apply” button is used instead of the “OK” button in the GUI windows.

### Validation of the Simulation Software

The simulation capability and functionality of the developed software for physiological conditions were validated at the level of single unit and whole system (Pham, 2000). First, the implementation of motoneuron model in the present simulation software was verified reproducing the non-linear firing behavior of the motoneuron under physiological conditions that has been shown in the XPPAUT software environment (Ermentrout, 2002). Second, the implementation of muscle unit model was verified reproducing the non-linear force production of the muscle under physiological conditions that has been demonstrated using the NEURON software (Hines and Carnevale, 1997). Lastly, for integration testing the non-linear input-output behaviors recently reported using the cat soleus motor unit model in the NEURON simulation environment were demonstrated using the present simulation software under the similar input conditions.

#### Non-linear Input–Output Behavior of a Cat Spinal Motoneuron

To verify the implementation of motoneuron model in the present software, our simulation results were directly compared with simulation results obtained in a previous study using XPPAUT under the same setup for the passive (i.e., R_N_ = 1.29 MΩ and τ_m_ = 7.2 ms at the soma and VA_SD_^DC^ = 0.76, VA_DS_^DC^ = 0.75 and VA_SD_^AC^ = 0.27 between the soma and all points over the dendrite at a similar path length of 0.6 mm from the soma) and active [i.e., I_Na,f_, I_K,dr_, I_Ca,N_, I_K(Ca)_, I_Na,p_ and calcium dynamics dependent E_Ca_ at the soma and I_Ca,L_ with constant E_Ca_ at the dendrite] membrane properties and input protocols (see Figures 2H,I in Kim et al., 2014 for values of model and input parameters).

The simulation was performed using the current version of the software through following steps: (1) select ‘Motoneuron’ from the drop-down MODEL menu in the Main window, (2) in the Model Parameters window check only ‘I_Naf_,’ ‘I_Nap_,’ ‘I_Kdr_,’ ‘I_K(Ca)_’ and ‘I_Can_’ for the SOMA and ‘I_Cal_’ and ‘constant calcium reversal potential’ for the DENDRITE and push “OK” or “Apply”, (3) in the Input Signals window select ‘Ramp,’ push “Generate,” and click “OK” or “Apply” on the ‘I_soma_’ tab for triangular current stimulation at the soma, (4) in the Output Signals window check ‘V’ and ‘Firing rate’ at the SOMA and ‘V’ at the DENDRITE along with ‘Overlapped’ display option and push “OK” or “Apply” and (5) push “Run” in the Main window. For the case of excitatory synaptic input without noise at the dendrite, ‘I_esyn_’ was additionally checked at the DENDRITE in the Model Parameters window and ‘Simulation Time’ was set to 20000 in the Simulation Conditions window. In the Input Signals window, ‘Peak Value’ for the ‘Ramp’ protocol was set to 0 on the ‘I_soma_’ tab for zero-current injection at the soma and the ‘Ramp’ protocol was selected setting ‘G_p_’ and ‘Duration’ to 0.12 and 10000 on the ‘I_syn_’ tab for triangular synaptic input at the dendrite. It should be noted that “OK” or “Apply” button must be pushed to reflect all changes that the user makes in individual sub-windows.

**FIGURE 6:**
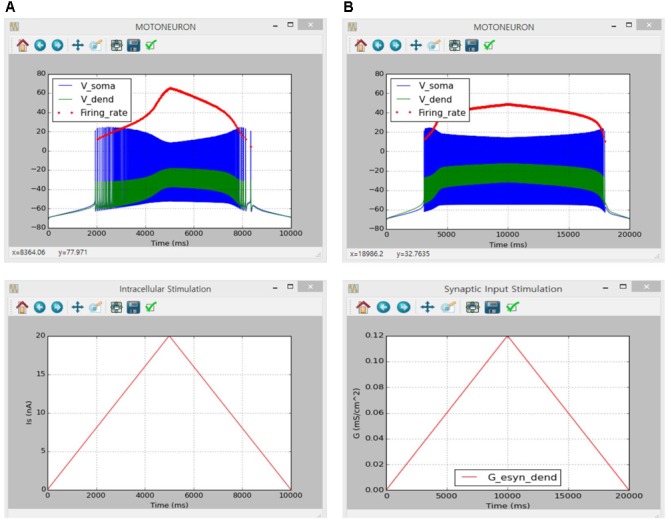
Non-linear input–output behavior of a cat spinal motoneuron. **(A)** Instantaneous firing rate (Firing rate, upper panel) and voltage responses at the soma (V_soma_, upper panel) and the dendrite (V_dend_, upper panel) against slowly ascending and descending current injected into the soma (I_S_, bottom panel). **(B)** Instantaneous firing rate (Firing rate, upper panel) and voltage responses at the soma (V_soma_, upper panel) and the dendrite (V_dend_, upper panel) against slowly ascending and descending variation in excitatory synaptic conductance at the dendrite (G, bottom panel).

**Figure 6** shows the simulation results of the counterclockwise hysteric firing output of the motoneuron in response to the triangular variation in the intracellular current injection at the soma (**Figure 6A**) and excitatory synaptic input without noise at the dendrite (**Figure 6B**). The results between the previous and current simulations were identical, indicating the capability of the current version of the software for physiological simulation of motoneuron behaviors experimentally observed (previous results are not shown but model code is available at ModelDB 239039).

#### Non-linear Input–Output Behavior of Cat Soleus Muscle

Similarly to the motoneuron case, the implementation of muscle unit model in the present software was verified directly comparing to simulation results obtained in a previous study using the NEURON software applying the same model parameters and input conditions (see Figures 4, 5 in Kim et al., 2015 for values of model and input parameters).

The simulation was performed using the current version of the software through following steps: (1) select ‘Muscle Fibers’ from the drop–down MODEL menu in the Main window, (2) in the Simulation Conditions window set ‘Simulation Time’ to 2000 and push “OK” or “Apply”, (3) in the Input Signals window check ‘Impulse Current,’ set ‘Frequency’ to 20, push “Generate,” and click “OK” or “Apply” for constant stimulation rate or check ‘Import Data,’ load ‘random_20hz.csv’ file from the I_axon_ folder, push “Generate,” and click “OK” or “Apply” for random stimulation rate on the ‘I_axon_’ tab, (4) in the Input Signals window check ‘Import Data,’ load ‘locomotor-like movement Xm.csv’ file from the Xm folder, push “Generate,” and click “OK” or “Apply” on the ‘X_m_’ tab, (5) in the Output Signals window check ‘X_CE_’, ‘X_m_’ and ‘F’ at the MODULE 3 along with ‘Overlapped’ display option and click “OK” or “Apply” and (6) push “Run” in the Main window.

**FIGURE 7:**
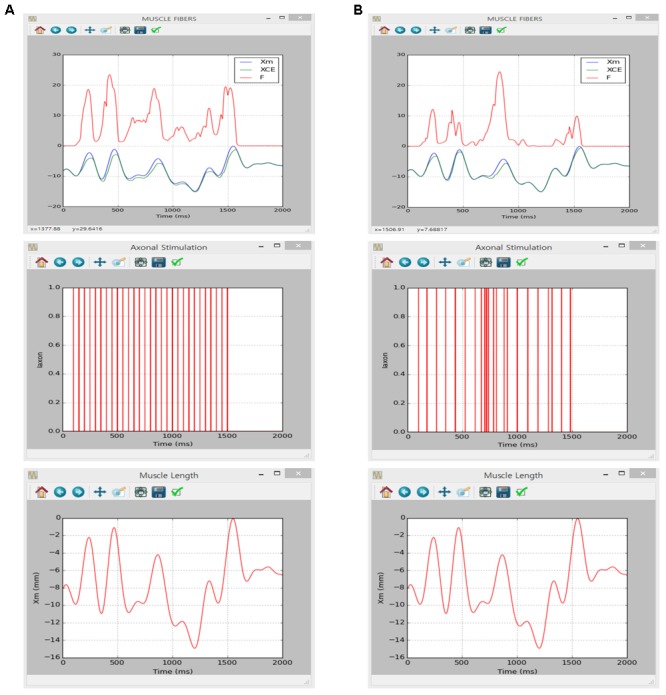
Non-linear input–output behavior of the cat soleus muscle. **(A)** Muscle force (F, upper panel) and length of the contractile element (X_CE_, upper panel) in response to a train of current impulses with a constant frequency of 20 Hz over the axon (I_axon_, middle panel) during locomotor-like movement (X_m_, bottom panel). **(B)** Muscle force (F, upper panel) and length of the contractile element (X_CE_, upper panel) in response to a train of current impulses with a random frequency with an average of 20 Hz over the axon (I_axon_, middle panel) during locomotor-like movement (X_m_, bottom panel).

**Figure 7** shows the movement dependent force output of the muscle unit model in response to the physiological stimulation rate (i.e., 20 Hz) without (**Figure 7A**) and with (**Figure 7B**) noise while dynamically varying the muscle length within the physiological range of locomotor-like movement. The results between the previous and current simulations in different software environments were identical, indicating the capability of the current version of the software for physiological simulation of muscle behaviors experimentally observed (previous results are not shown but model code is available at ModelDB 235769).

#### Non-linear Input-Output Behavior of a Cat Slow Motor Unit

The same motoneuron and muscle unit models verified in the previous sections (Non-linear Input—Output Behavior of a Cat Spinal Motoneuron and Non-linear Input–Output Behavior of Cat Soleus Muscle) were integrated into the motor unit model to validate that the current software met the simulation requirements set for the motor unit system in this study. The results obtained in a previous study using an anatomically reconstructed motoneuron in the NEURON software environment were reproduced in the present software environment under the similar input protocols used in the previous study (see Figures 3–6 in Kim, 2017b).

The simulation was performed using the current version of the software through following steps: (1) select ‘Motor Unit’ from the drop-down MODEL menu in the Main window, (2) in the Model Parameters window check only ‘I_Naf_,’ ‘I_Nap_,’ ‘I_Kdr_,’ ‘I_K(Ca)_’ and ‘I_Can_’ for the SOMA and ‘I_Cal_’ and ‘constant calcium reversal potential’ for the DENDRITE on the ‘Motoneuron’ tab, set ‘P_0_’ to 1 for the MODULE 3 on the ‘Muscle Fibers’ tab, and push “OK” or “Apply,” (3) in the Input Signals window select ‘Step’ or ‘Ramp’ and push “Generate” on the ‘I_soma_’ tab for current stimulation at the soma, check ‘Isometric’ and push “Generate” on the ‘X_m_’ tab for muscle length condition, and click “OK” or “Apply”, (4) in the Output Signals window check ‘V’ and ‘Firing rate’ at the SOMA on the ‘Motoneuron’ tab and ‘F’ at the MODULE 3 on the ‘Muscle Fibers’ tab and push “OK” or “Apply” and (5) push “Run” in the Main window. For the case of excitatory synaptic input without noise over the dendrite, ‘I_esyn_’ was additionally checked at the DENDRITE in the Model Parameters window and ‘Peak Value’ for the ‘Ramp’ protocol was set to 0 on the ‘I_soma_’ tab for zero-current injection at the soma. Under the ‘Ramp’ synaptic input condition, ‘Simulation Time’ was set to 20000 in the Simulation Conditions window and ‘G_p_’ and ‘Duration’ were set to 0.12 and 10000 for the DENDRITE on the ‘I_syn_’ tab in the Input Signals window.

**FIGURE 8:**
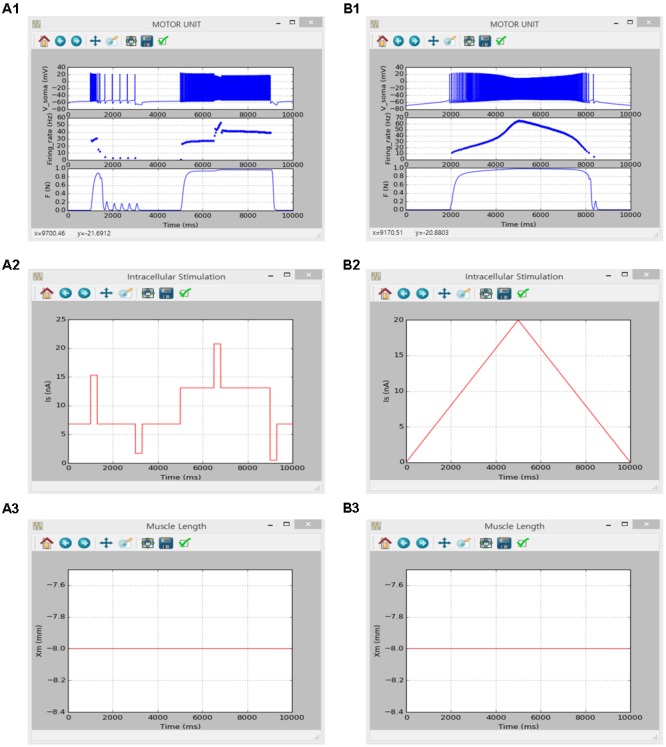
Non-linear input-output behavior of a slow motor unit with I_soma_. **(A1–A3)** Voltage responses at the soma (V_soma_, upper panel in **A1**), instantaneous firing rate (Firing_rate, middle panel in **A1**) and muscle force (F, bottom panel in **A1**) in response to the step current with alternating excitatory and inhibitory pulses injected at the soma (I_s_, **A2**) at the optimal muscle length (X_m_, **A3**). **(B1–B3)** Voltage responses at the soma (V_soma_, upper panel in **B1**), instantaneous firing rate (Firing_rate, middle panel in **B1**) and muscle force (F, bottom panel in **B1**) against slowly ascending and descending current injections at the soma (I_S_, **B2**) while maintaining the optimal muscle length (X_m_, **B3**).

**FIGURE 9:**
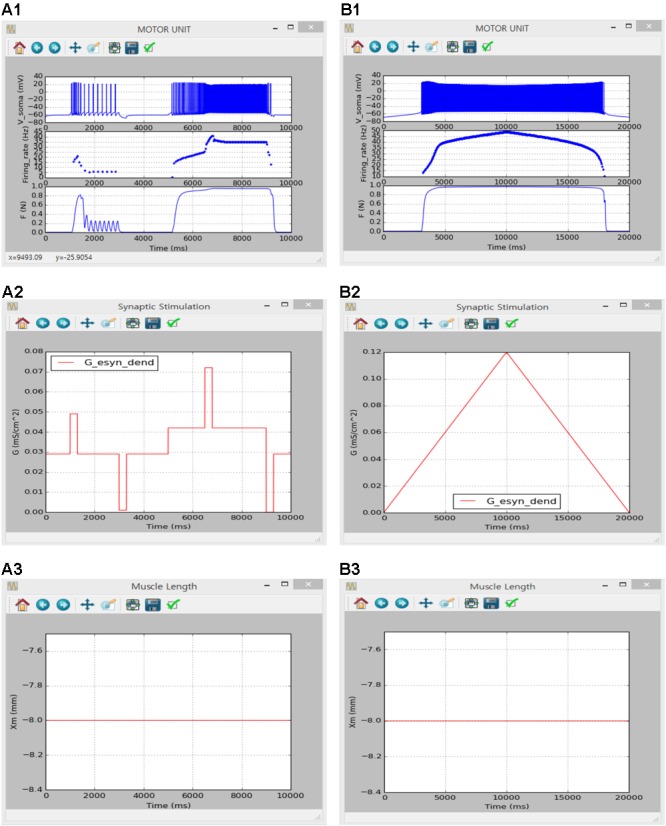
Non-linear input-output behavior of a slow motor unit with G_esyn,dend_. **(A1–A3)** Voltage responses at the soma (V_soma_, upper panel in **A1**), instantaneous firing rate (Firing_rate, middle panel in **A1**) and muscle force (F, bottom panel in **A1**) in response to the step variation with alternating excitatory and inhibitory pulses in excitatory synaptic conductance at the dendrite (G_esy,dend_, **A2**) at the optimal muscle length (X_m_, **A3**). **(B1–B3)** Voltage responses at the soma (V_soma_, upper panel in **B1**), instantaneous firing rate (Firing_rate, middle panel in **B1**) and muscle force (F, bottom panel in **B1**) against slowly ascending and descending variation in excitatory synaptic conductance at the dendrite (G_esy,dend_, **B2**) while maintaining the optimal muscle length (X_m_, **B3**).

**Figures 8, 9** show the firing history and muscle length dependent force production of the motor unit model in response to alternating step and triangular variation in the intracellular current injected into the soma (**Figure 8**) and excitatory synaptic input without noise at the dendrites (**Figure 9**) while holding the muscle length constant at the optimal length. Directly comparing the current and previous simulations was difficult because of the differences in the motoneuron model between the two cases. However, the current simulation software could demonstrate similar non-linear input–output behaviors of the motor unit system as shown in the previous simulations (previous results are not shown but model code is available at ModelDB 235769).

The motor unit model validated in **Figures 8, 9** was extended by adding additional active currents to both the soma (i.e., I_H_ along with constant E_Ca_) and the dendrite [i.e., I_Na,f_, I_K,dr_, I_Ca,N_, I_K(Ca)_, I_Na,p_ and I_H_] and further validated reproducing the results from a previous study performed in the NEURON software environment for the triangular variation in inhibitory synaptic inputs with noise at the soma and excitatory synaptic inputs with noise at the dendrites for the optimal muscle length (see Figures 6, 7 in Powers et al., 2012).

The simulation was performed using the current version of the software through following steps: (1) select ‘Motor Unit’ from the drop-down MODEL menu in the Main window, (2) in the Model Parameters window check only ‘I_Naf_,’ ‘I_Nap_,’ ‘I_Kdr_,’ ‘I_K(Ca)_,’ ‘I_Can_,’ ‘I_H_,’ ‘constant calcium reversal potential’ and ‘I_isyn_’ for the SOMA and ‘I_Cal_,’ ‘I_Naf_,’ ‘I_Nap_,’ ‘I_Kdr_,’ ‘I_K(Ca)_,’ ‘I_Can_,’ ‘I_H_,’ ‘constant calcium reversal potential’ and ‘I_esyn_’ for the DENDRITE on the ‘Motoneuron’ tab, set ‘P_0_’ to 1 for the MODULE 3 on the ‘Muscle Fibers’ tab, and push “OK” or “Apply,” (3) in the Simulation Conditions window set ‘Simulation Time’ to 20000 and push “OK” or “Apply,” (4) in the Input Signals window set ‘I_0_’ and ‘scale factor’ to 0 and push “Generate” on the ‘I_soma_’ tab for zero-current injection at the soma, select ‘Ramp’ with ‘Duration’ of 10000 checking ‘Noise’ at both SOMA and DENDRITE for the push-pull input condition and push “Generate” on the ‘I_syn_’ tab, check ‘Isometric’ and push “Generate” on the ‘X_m_’ tab for muscle length condition, and click “OK” or “Apply”, (5) in the Output Signals window check ‘V’ and ‘Firing rate’ at the SOMA on the ‘Motoneuron’ tab and ‘F’ at the MODULE 3 on ‘Muscle Fibers’ tab and push “OK” or “Apply”, and (6) push “Run” in the Main window. Under the proportional inhibition input condition, ‘G_0_’ and ‘G_p_’ for the ‘Ramp’ protocol at the SOMA were set to 0.03 and 0.13 on the ‘I_syn_’ tab in the Input Signals window.

**FIGURE 10:**
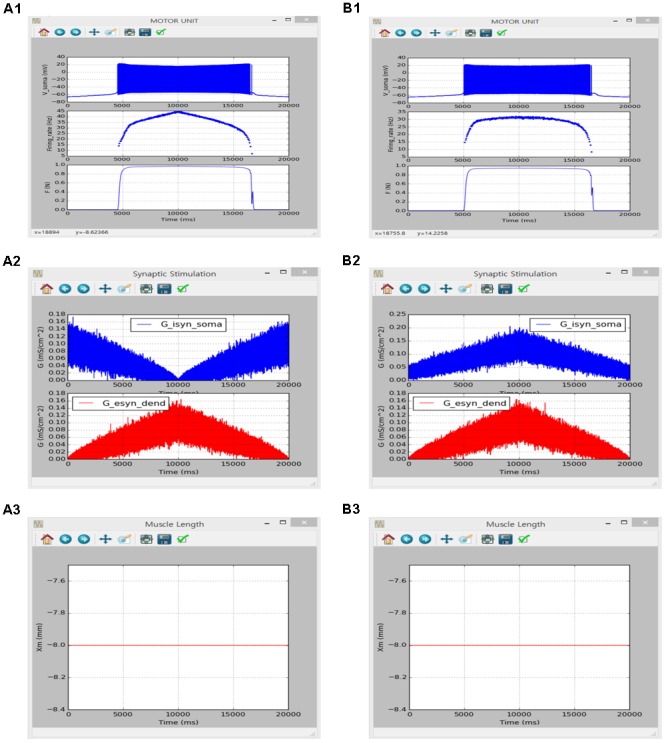
Non-linear input-output behavior of a slow motor unit with G_isyn,soma_ and G_esyn,dend_ including intensity-dependent synaptic noise. **(A1–A3)** Voltage responses at the soma (V_soma_, upper panel in **A1**), instantaneous firing rate (Firing_rate, middle panel in **A1**) and muscle force (F, bottom panel in **A1**) against triangular variation in inhibitory synaptic input at the soma (G_isyn,soma_, blue lines in **A2**) and excitatory synaptic input at the dendrite (G_esyn,dend_, red lines in **A2**) in a push-pull manner at the optimal muscle length (X_m_, **A3**). **(B1–B3)** Voltage responses at the soma (V_soma_, upper panel in **B1**), instantaneous firing rate (Firing_rate, middle panel in **B1**) and muscle force (F, bottom panel in **B1**) against triangular variation in inhibitory synaptic input at the soma (G_isyn,soma_, blue lines in **B2**) and excitatory synaptic input at the dendrite (G_esyn,dend_, red lines in **B2**) in a proportional manner while maintaining the optimal muscle length (X_m_, **B3**).

**Figure 10** shows the influence of synaptic input organization on the firing behavior of the motoneuron and resultant force production of the muscle unit in the motor unit model under push-pull (**Figure 10A**) and proportional inhibition (**Figure 10B**) conditions while holding the muscle length constant at the optimal length. Although performing a quantitative comparison was difficult due to the use of a different modeling approach in the previous study, the results of the previous and current simulations were qualitatively similar, confirming the capability of the current version of the software for physiological simulation of the motor unit system under noisy synaptic inputs at the soma and the dendrite (previous results are not shown but model code is available at ModelDB 143671).

## Discussion

In this study, we constructed a computationally efficient physiologically plausible model of a motor unit and developed Python-based simulation software that enables virtual experiments investigating motor unit behavior. The design and implementation of the simulation software allow for a single motoneuron, muscle unit and motor unit to be simulated and analyzed individually over the full physiological range of inputs, including intracellular current injections at the soma and synaptic inputs to both the soma and dendrite in the motoneuron, current impulse stimulation over the axonal nerve and length variation under isometric, isokinetic and dynamic conditions in the muscle unit. The pure GUI-based simulation software developed in this study may provide an efficient tool not only for systematic investigations of non-linear motor unit behavior but also for educators and students of neuromuscular physiology underlying force generation and control at a cellular level.

Most previous simulation software for neuromuscular systems have been developed separately for nervous and muscular systems. Simulation software mostly used for investigations of single neurons, such as NEURON and GENESIS, were developed to enable the anatomical construction of neuron dendrites. However, in anatomically reconstructed motoneurons, simultaneously modulating multiple cell-type-specific properties, including input resistance and membrane time constant at the soma and signal propagation properties over the dendrites, which are the essential properties underlying the dendritic excitability and firing output, is challenging (Kim, 2017a). The new reduced modeling approach used in this study can overcome this limitation of anatomical modeling approaches by providing an efficient way to capture the cell-type-specific electrical properties in an analytical manner (Powers and Heckman, 2017).

Most simulation software for skeletal muscles have employed Hill-type muscle models likely due to their computational efficiency and simplicity (Delp et al., 2007; Sartori and Farina, 2016). However, Hill-type muscle models are well known to be suited for isometric contractions under full-excitation conditions (Sandercock and Heckman, 1997; Millard et al., 2013). In this study, this range limitation of two physiological inputs (i.e., length variation and stimulation rate) to the muscle unit was extended by applying the recently developed muscle modeling approach. In the current version of the simulation software, the muscle unit behavior can be simulated over the full physiological range of length variations and stimulation rates under various conditions that can be experimentally established (see Stimulation Protocols for Motoneurons and Variation in Muscle Length in the Materials and Methods).

The simulation software developed in this study may provide a user-friendly environment for the feedforward input–output analysis of a single motoneuron, muscle unit and motor unit. Through this simulation and analysis, several fundamental issues in the field of motor neuroscience may be efficiently investigated under length variation including synaptic input organization over the motoneuron for proper force production in the muscle unit, input–output transfer characteristics of motor unit system and control strategy of motor unit functions. These studies would contribute to advancing our understanding of neural coding mechanism for desired motor outputs and facilitating the development of direct neural-machine interface over a wide range of behaviors.

Since the motor unit system is well known to be a quantal element underlying movements in all animals, the new modeling approach and simulation software developed in this study could also be applied for physiological modeling of the motor unit in worms (Szigeti et al., 2014). The capability of our spike-driven muscle unit model for capturing force production over a wide range of length variation (i.e., isometric, isokinetic and dynamic) and stimulation rate (i.e., twitch, sub-tetanic and tetanic) may provide a useful basis for physiological modeling of locomotion in worms (Boyle and Cohen, 2008). In addition, the modular modeling framework may allow model parameters to be determined for individual sub-modules based directly on experimental data. Likewise, our reduced modeling approach for the motoneuron may also be useful in uniquely determining model parameter values to retain the fundamental system properties including somatic input resistance, membrane time constant and electrotonical signal propagation of the dendrites that are experimentally measurable from real cells (Vella et al., 2013). Furthermore, the simulation software developed in this study may provide an efficient tool for investigation of fundamental issues related to neural control of muscle force in worms.

Furthermore, several features of the developed simulation software may also allow it to be an educational tool that efficiently demonstrates and explains physiological principles of the neuromuscular mechanisms underlying biological movements. First, the fundamental building blocks of neuromuscular systems, i.e., single motoneurons, muscle fibers and their connected form, which is referred to as a motor unit, can be modeled and simulated in a hierarchical manner. Second, the electrical activities of motoneurons and the mechanical behaviors of muscle fibers can be modeled and simulated based on biophysically plausible, physiologically realistic mechanisms to achieve biological realism. Third, the input conditions for the simulations of the selected model can be customized to demonstrate a variety of experimental observations reported in the literature. Finally, an intuitive graphical user interface could make it easier to adjust the parameter values of the models and input signals to reflect a wide range of physiological conditions, display the simulation results of the selected model variables during the simulation, and save the files for offline data analysis.

However, the current version of the simulation software must be further improved to achieve biological realism and better performance. First, the two-compartment modeling approach used to model the motoneuron must be extended to reflect the influence of the complex dendritic structure on the activation of persistent inward currents over the dendrites. Second, the non-linear muscle properties, such as force potentiation, force decline (or sag) during unfused tetanic contractions and velocity-dependent force development, should be included to allow for simulations of different types of motor units. Third, feedback signals from the muscle spindle should be added to allow for simulations of a muscle unit while the muscle length varies over time. Fourth, the biophysically plausible axon model should be incorporated to realistically simulate the transduction of neural signals from the motoneuron to the muscle unit. Fifth, software modules should be added to process the simulation data according to the purposes of the analysis. Sixth, the present software code was optimized by employing the numpy data type for the interpolation function and the blitting technique to make animations efficient in Matplotlib by only redrawing the plot elements that are changing at each frame. However, the performance of the simulation software is expected to be further improved by implementing the numerical integration of models with Cython (Behnel et al., 2011). Lastly, in this study the software was designed for simulations of a specific model rather than a fully flexible tool. The flexibility of the current version of the simulation software operating only though GUI could be greatly improved by adding the script-mode where the model class can be edited by the user (see **Figure 4**).

## Conclusion

The simulation software developed in this study may provide an efficient tool for investigations of the neural mechanisms underlying force control. In addition, we hope that the simulation software, which enables the simulation of motor unit systems at the cellular level, could be useful not only to the instructors for effective teaching but also to students for practice and research outside the formal curriculum.

## Author Contributions

HK conceived the study, modeled the motor unit system, analyzed the requirements for simulation software, and wrote and revised the manuscript. HK and MK designed and tested the simulation software. MK implemented and documented the simulation software.

## Conflict of Interest Statement

The authors declare that the research was conducted in the absence of any commercial or financial relationships that could be construed as a potential conflict of interest.

## Footnotes

1 www.omg.org/spec/UML/2.1.2

2 https://github.com/NMSL-DGIST/PyMUS
