# Supplementary material for: PyMUS: Python-Based Simulation Software for Virtual Experiments on Motor Unit System
Source: Front Neuroinform. 2018 Apr 11;12:15. doi: 10.3389/fninf.2018.00015 (PMC5904262; doi:10.3389/fninf.2018.00015)
Supplement: Supplementary file 1 [file Data_Sheet_1.docx]

**Appendix**

The modeling procedure including the equation derivation and parameter setting was fully presented in our previous studies for the motoneuron (Kim et al., 2014) and the muscle (Kim et al., 2015).

**1. System equations used for the reduced motoneuron model**

$C_{m,S}\cdot\frac{dV_{S}}{dt}=-\sum I_{soma}-G_{m,S}\cdot\left(V_{S}-E_{Leak,S}\right)-\frac{G_{C}}{p}\cdot\left(V_{S}-V_{D}\right)+I_{S}$ (1.1)

$$\sum I_{soma}=I_{Naf,S}+I_{Kdr,S}+I_{Can,S}+I_{K(Ca),S}+I_{Nap,S}+I_{H,S}+I_{syn,S}$$

$C_{m,D}\cdot\frac{dV_{D}}{dt}=-\sum I_{dendrite}-G_{m,D}\cdot\left(V_{D}-E_{Leak,D}\right)-\frac{G_{C}}{1-p}\cdot\left(V_{D}-V_{S}\right)$ (1.2)

$$\sum I_{dendrite}=I_{Naf,D}+I_{Kdr,D}+I_{Can,D}+I_{K(Ca),D}+I_{Nap,D}+I_{H,D}+I_{Cal,D}+I_{syn,D}$$

where the subscripts *S* and *D* indicate the soma and dendrites, respectively, *V* is the membrane potential, *E_Leak_* is the reversal potential of the leak current, *G* and *C* indicate specific membrane conductance and capacitance, ∑*I* indicates transmembrane currents and *I_S_* is intracellularly injected current at the soma.

1.1 *Inverse equations for the five cable parameters*

$G_{m,S}=\frac{1-VA_{DS}^{DC}}{r_{N}(1-VA_{SD}^{DC}VA_{DS}^{DC})}$ (1.3)$G_{m,D}=\frac{pVA_{\text{DS}}^{\text{DC}}\text{(1}-VA_{SD}^{DC})}{(1-p)r_{N}VA_{SD}^{DC}(1-VA_{SD}^{DC}VA_{DS}^{DC})}$ (1.4)$G_{C}=\frac{pVA_{\text{DS}}^{DC}}{r_{N}(1-VA_{SD}^{DC}VA_{DS}^{DC})}$ (1.5)$C_{m,D}=\frac{1}{\omega(1-p)}\sqrt{\frac{G_{C}^{2}}{\left(VA_{SD}^{AC}\right)^{2}}-\left\{G_{C}+G_{m,D}(1-p)\right\}^{2}}$ (1.6)$C_{m,S}=\frac{\tau_{m}\left\{p\left(1-p\right)\tau_{m}G_{m,S}G_{m,D}+pG_{m,S}\left(\tau_{m}G_{C}-C_{m,D}\right)+p^{2}G_{m,S}C_{m,D}+\left(1-p\right)\left(\tau_{m}G_{C}G_{m,D}-G_{C}C_{m,D}\right)\right\}}{p\left\{\left(1-p\right)\left(\tau_{m}G_{m,D}-C_{m,D}\right)+\tau_{m}G_{C}\right\}}$(1.7)

where *VA* indicates the voltage attenuation factor calculated between the soma and dendrites and *r_N_* is the value of R_N_ normalized to the surface area of the somatic compartment (see (Kim et al., 2009;Kim and Jones, 2012) for derivation of the inverse equations (1.3)-(1.7)). In the current version of PyMUS (PyMUS v2.0), the surface area for the somatic compartment, *p* and *ω* were set to 0.3157 m^2^, 0.5 and 2π×250 Hz, respectively (see Figure 2 in (Kim et al., 2014) for the parameter setting).

1.2 *Equations for the active currents*

In the current version of PyMUS (PyMUS v2.0), the soma and dendrite have the same types of active membrane properties, except for the additional inclusion of low voltage activated L-type calcium current in the dendrite. All voltage gated ion channels were modeled based on the HH type formulation as follows: *I_Ion_*=*G_Ion_*·*m_ion_^a^*·*h_ion_^b^*·(*V*-*E_Ion_*), where *G_Ion_* is the peak conductance of the specific ion current, *m_ion_* and *h_ion_* are the gating variables for activation and inactivation, *a* and *b* are the order of activation and inactivation, and *E_Ion_* is the reversal potential for the ion of interest. The active mechanisms included in both the soma and the dendrite were indicated by the subscript *X* unless otherwise stated with the subscript *S* or *D* in the following equations.

Fast Na^+^ current

$I_{Naf,X}=G_{Naf,X}\cdot m_{naf}^{3}\cdot h_{naf}\cdot\left(V_{X}-E_{Na,X}\right)$ (1.8)

$\frac{dm_{naf}}{dt}=\alpha_{m}\cdot\left(1-m_{naf}\right)-\beta_{m}\cdot m_{naf}$where

$\alpha_{m}=\frac{\alpha_{nafm1,X}\cdot\left(V_{X}+\alpha_{nafm2,X}\right)}{\exp\left(-\frac{V_{X}+\alpha_{nafm2,X}}{\alpha_{nafm3,X}}\right)+\alpha_{nafm4,X}}$, $\beta_{m}=\frac{\beta_{nafm1,X}\cdot\left(V_{X}-\beta_{nafm2,X}\right)}{\exp\left(\frac{V_{X}-\beta_{nafm2,X}}{\beta_{nafm3,X}}\right)+\beta_{nafm4,X}}$

$\frac{dh_{naf}}{dt}=\frac{h_{\infty}-h_{naf}}{\tau_{h}}$ where

$h_{\infty}=\frac{1}{1+\exp\left(\frac{V_{X}-\gamma_{nafh1,X}}{\gamma_{nafh2,X}}\right)}$, $\tau_{h}=\frac{\gamma_{nafh6,X}}{\exp\left(\frac{V_{S}-\gamma_{nafh3,X}}{\gamma_{nafh4,X}}\right)+\exp\left(-\frac{V_{S}-\gamma_{nafh3,X}}{\gamma_{nafh5,X}}\right)}$

Delayed rectifier K^+^ current

$I_{Kdr,X}=G_{Kdr,X}\cdot n_{kdr}^{4}\cdot(V_{X}-E_{K,X})$ (1.9)

$\frac{d(n_{kdr})}{dt}=\frac{(n_{\infty}-n_{kdr})}{\tau_{n}}$where

$n_{\infty}=\frac{1}{1+\exp\left(-\frac{V_{X}-\gamma_{kdrm1,X}}{\gamma_{kdrm2,X}}\right)}$, $\tau_{n}=\frac{\gamma_{kdrm6,X}}{\exp\left(\frac{V_{X}-\gamma_{kdrm3,X}}{\gamma_{kdrm4,X}}\right)+\exp\left(-\frac{V_{X}-\gamma_{kdrm3,X}}{\gamma_{kdrm5,X}}\right)}$

N-Type Ca^2+^ current

$I_{Can,X}=G_{Can,X}\cdot m_{can}^{2}\cdot h_{can}\cdot(V_{X}-E_{Ca,X})$ (1.10)

$\frac{d(m_{can})}{dt}=\frac{m_{\infty}-m_{can}}{\tau_{m}}$ where $m_{\infty}=\frac{1}{1+\exp\left(-\frac{V_{X}-\gamma_{canm1,X}}{\gamma_{canm2,X}}\right)}$, $\tau_{m}=\gamma_{canm3,X}$

$\frac{d(h_{can})}{dt}=\frac{h_{\infty}-h_{can}}{\tau_{h}}$ where $h_{\infty}=\frac{1}{1+\exp\left(\frac{V_{X}-\gamma_{canh1,X}}{\gamma_{canh2,X}}\right)}$, $\tau_{h}=\gamma_{canh3,X}$

Ca^2+^ concentration dynamics

$\frac{d[Ca^{2+}]_{i,X}}{dt}=f_{X}(-\alpha_{X}\cdot I_{Ca,X}-K_{Ca,X}\cdot[Ca^{2+}]_{i,X})$ (1.11)

Equilibrium potential for Ca^2+^

$E_{Ca,X}=\frac{1000\cdot R\cdot T}{Z_{Ca}\cdot F}\cdot\log\left(\frac{[Ca^{2+}]_{o,X}}{[Ca^{2+}]_{i,X}}\right)-70$ (1.12)

where *R*=8.31441 VC/mol·K, *T*=309.15 K, *Z_Ca_*=2, *F*=96485.309 C/mol with [*Ca^2+^*]*_o_*=2 mM.

Calcium dependent K+ current

$I_{K(Ca),X}=G_{K(Ca),X}\cdot\frac{[Ca^{2+}]_{i,X}}{[Ca^{2+}]_{i,X}+K_{d,X}}\cdot(V_{X}-E_{K,X})$ (1.13)

Persistent Na^+^ current:

$I_{Nap,X}=G_{Nap,X}\cdot m_{nap}^{3}\cdot(V_{X}-E_{Na,X})$ (1.14)

$\frac{d(m_{nap})}{dt}=\alpha_{m}\cdot\left(1-m_{nap}\right)-\beta_{m}\cdot m_{nap}$ where

$\alpha_{m}=\frac{\alpha_{napm1,X}\cdot(V_{X}-\alpha_{napm2,X})}{\exp\left(-\frac{V_{X}-\alpha_{napm2,X}}{\alpha_{napm3,X}}\right)+\alpha_{napm4,X}}$, $\beta_{m}=\frac{\beta_{napm1,X}\cdot(V_{X}-\beta_{napm2,X})}{\exp\left(\frac{V_{X}-\beta_{napm2,X}}{\beta_{napm3,X}}\right)+\beta_{napm4,X}}$

Hyperpolarization-activated mixed cation current

$I_{H,X}=G_{H,X}\cdot m_{h}\cdot(V_{X}-E_{H,X})$ (1.15)

$\frac{d(m_{h})}{dt}=\frac{m_{\infty}-m_{h}}{\tau_{m}}$ where $m_{\infty}=\frac{1}{\exp\left(\frac{V_{s}+\gamma_{hm1,X}}{\gamma_{hm2,X}}\right)}$, $\tau_{m}=\gamma_{hm3,X}$

Synaptic current

$I_{syn,X}=I_{esyn,X}+I_{isyn,X}$ (1.16)

$$I_{esyn,X}=G_{esyn,X}\cdot\left(V_{X}-E_{esyn,X}\right)$$

$$\frac{dG_{esyn,X}}{dt}=-\frac{1}{\tau_{esyn,X}}\cdot(G_{esyn,X}-G_{esyn0,X})+\sqrt{\frac{2\sigma_{esyn,X}^{2}}{\tau_{esyn,X}}}\cdot\chi_{1}$$

$$I_{isyn,X}=G_{isyn,X}\cdot\left(V_{X}-E_{isyn,X}\right)$$

$$\frac{dG_{isyn,X}}{dt}=-\frac{1}{\tau_{isyn,X}}\cdot(G_{isyn,X}-G_{isyn0,X})+\sqrt{\frac{2\sigma_{isyn,X}^{2}}{\tau_{isyn,X}}}\cdot\chi_{2}$$

Dynamical variation in excitatory (*G_esyn,X_*) and inhibitory (*G_isyn,X_*) synaptic conductance with noise was formulated using the Ornstein-Uhlenbeck process, where *G_esyn0,X_* and *G_isyn0,X_* are the mean of *G_esyn,X_* and *G_isyn,X_*, *τ* is a time constant, *σ* is the standard deviation from the mean conductance and *χ* is a random Gaussian noise process with a mean of 0 and a standard deviation of 1 (Destexhe et al., 2001).

Low voltage activation L-type Ca^2+^ current

$I_{Cal,D}=G_{Cal,D}\cdot l_{cal}\cdot(V_{D}-E_{Ca,D})$ (1.17)

$\frac{dl_{cal}}{dt}=\frac{l_{\infty}-l_{cal}}{\tau_{l}}$ where $l_{\infty}=\frac{1}{1+\exp\left(-\frac{V_{D}-\gamma_{calm1,D}}{\gamma_{calm2,D}}\right)}$, $\tau_{l}=\gamma_{calm3,D}$

The equations for *I_Naf_*, *I_Kdr_*, *I_CaN_*, *I_Nap_*, *I_CaL_* and *E_Ca_* were adopted from (McIntyre and Grill, 2002), *d[Ca^2+^]_i_/dt* and *I_K(Ca)_* were from (Booth et al., 1997), and *I_H_* was from (Powers et al., 2012).

**2. System equations used for the muscle-tendon model**

Module 1: The transformation of action potentials to calcium dynamics in the sarcoplasm

$\frac{d\left[Ca_{SR}\right]}{dt}=-K1\cdot CS_{0}\cdot\left[Ca_{SR}\right]+\left(K1\cdot\left[Ca_{SR}\right]+K2\right)\cdot\left[Ca_{SR}CS\right]-R+U$ (2.1)

$\frac{d\left[Ca_{SR}CS\right]}{dt}=K1\cdot CS_{0}\cdot\left[Ca_{SR}\right]-\left(K1\cdot\left[Ca_{SR}\right]+K2\right)\cdot\left[Ca_{SR}CS\right]$ (2.2)

where [*Ca_SR_*], [*Ca_SR_CS*] and *CS_0_* indicate the concentration of free calcium ions, Ca^2+^ bound to calsequestrin and total calsequestrin in the sarcoplasmic reticulum (*SR*), respectively, *K1* and *K2* are the forward and backward constants for reaction kinetics between the Ca_SR_ and Ca_SR_CS and the release (*R*) of Ca^2+^ from the SR and the uptake (*U*) of Ca^2+^ into the SR were mathematically modeled as

$R=\left[Ca_{SR}\right]\cdot R_{\max}\cdot\sum_{i=1}^{n} \left(1-\exp\left(-\frac{t-t_{i}}{\tau_{1}}\right)\right)\cdot\exp\left(-\frac{t-t_{i}}{\tau_{2}}\right)$,

$$U=U_{\max}\cdot\left(\frac{\left[Ca_{SP}\right]^{2}\cdot K^{2}}{1+\left[Ca_{SP}\right]\cdot K+\left[Ca_{SP}\right]^{2}\cdot K^{2}}\right)^{2}$$

$\frac{d\left[Ca_{SP}\right]}{dt}=-\left(K3\cdot B_{0}+K5\cdot T_{0}\right)\cdot\left[Ca_{SP}\right]+\left(K3\cdot\left[Ca_{SP}\right]+K4\right)\cdot\left[Ca_{SP}B\right]+\left(K5\cdot\left[Ca_{SP}\right]+K6\right)\cdot\left[Ca_{SP}T\right]+R-U$(2.3)

$\frac{d\left[Ca_{SP}B\right]}{dt}=K3\cdot B_{0}\cdot\left[Ca_{SP}\right]-\left(K3\cdot\left[Ca_{SP}\right]+K4\right)\cdot\left[Ca_{SP}B\right]$ (2.4)

$\frac{d\left[Ca_{SP}T\right]}{dt}=K5\cdot T_{0}\cdot\left[Ca_{SP}\right]-\left(K5\cdot\left[Ca_{SP}\right]+K6\right)\cdot\left[Ca_{SP}T\right]$ (2.5)

where [*Ca_SP_*], [*Ca_SP_B*], [*Ca_SP_T*], *B_0_* and *T_0_* indicate the concentration of free calcium ions, Ca^2+^ bound to free calcium-buffering proteins (*B*), Ca^2+^ bound to troponin (*T*), total free calcium-buffering proteins and total troponin in the sarcoplasm (*SP*), respectively and *K3*-*K6* are the rate constants for chemical reactions between the *Ca_SP_*, *B*, *T*, *Ca_SP_B* and *Ca_SP_T* in which *K5* and *K6* were modulated as a function of muscle length (*X_m_*) and activation level ($\tilde{A}$) under steady Ca^2+^ stimulation as follows,

$K5=K5_{i}\cdot\phi(X_{m}), \left\{\begin{matrix} \phi(X_{m})=\phi_{1}\cdot X_{m}+\phi_{2}, for X_{m} < optimal length \\ \phi(X_{m})=\phi_{3}\cdot X_{m}+\phi_{4}, for X_{m }\geq optimal length \end{matrix}\right.$,

$$K6=\frac{K6_{i}}{1+5\cdot\tilde{A}}$$

In the current version of PyMUS (PyMUS v2.0), *CS_0_*, *B_0_* and *T_0_* were set to 30 (mM), 0.43 (mM) and 70 (µM), respectively (see the Methods in (Kim et al., 2015) for the parameter setting).

Module 2: The transformation of the sarcoplasmic calcium dynamics to muscle activation dynamics

(2.6)

where *V_m_* is the time derivative of *X_m_* and the $\tilde{A}\left(t\right)$ and its exponent (*α*(*t*)) were mathematically modeled applying the Morris-Lecar formulation that has been used to mathematically represent the dynamics of gating variables underlying membrane excitability (Morris and Lecar, 1981),

where $\tilde{A}_{\infty}=0.5\cdot\left(1+\tanh\frac{\left[Ca_{SP}T\right]/T_{0}-C1}{C2}\right)$, $\tau_{\tilde{A}}=C3\cdot\left(\cosh\frac{\left[Ca_{SP}T\right]/T_{0}-C4}{2\cdot C5}\right)^{-1}$

$$\alpha(t)=\left\{\begin{matrix} \alpha for isometric and isokinetic contractio n \\ \alpha+\alpha_{1}\cdot\left(1+\tanh\frac{t-\alpha_{2}}{\alpha_{3}}\right) for dynamic contractio n \end{matrix}\right.$$

In the current version of PyMUS (PyMUS v2.0), *α* and *α*_1_– *α*_3_ were set to 2, 4.77, 400 (ms) and 160 (ms), respectively. The *β* and γ were set to 0.47 and 0.001 (s/mm) for lengths shorter than the optimal length during muscle lengthening under dynamic contraction condition otherwise both were set to 0 (see Table 1 in (Kim et al., 2015) for the parameter setting).

Module 3: The transformation of muscle activation to muscle force

$F=P_{0}\cdot K_{SE}\cdot(\Delta X_{m}-\Delta X_{CE})$ (2.7)

where *P_0_* is the peak force at the optimal length under full excitation in the isometric condition, *K_SE_* is the stiffness of the serial element normalized by *P_0_* and the length (*X_CE_*) of contractile element was calculated using the modified Hill-Mashma equations along with the length-tension relationship (*g*(*X_m_*)),

$$\frac{dX_{CE}}{dt}=\frac{-b_{0}\cdot\left(P_{0}\cdot g(X_{m})\cdot A(t)-F\right)}{F+a_{0}\cdot g(X_{m})\cdot A(t)} , for F\leq P_{0}\cdot g(X_{m})\cdot A(t)$$

$$\frac{dX_{CE}}{dt}=\frac{-d_{0}\cdot\left(P_{0}\cdot g(X_{m})\cdot A(t)-F\right)}{2\cdot P_{0}\cdot g(X_{m})\cdot A(t)-F+c_{0}\cdot g(X_{m})\cdot A(t)} , for F>P_{0}\cdot g(X_{m})\cdot A(t)$$

$$g(X_{m})=\exp\left\{-\left(\frac{X_{m}-g_{1}}{g_{2}}\right)^{2}\right\}$$

In the current version of PyMUS (PyMUS v2.0), *a_0_* and *c_0_* determined from *P_0_*, length-tension and velocity-tension relationship for entire muscle were scaled by multiplying the ratio of new to default *P_0_* for simulations of muscle units that may show various levels of *P_0_*.
